# Allogeneic hematopoietic stem cell transplantation remains a feasible approach for elderly with acute myeloid leukemia: a 10-year experience

**DOI:** 10.1007/s00277-023-05226-1

**Published:** 2023-04-19

**Authors:** Katarzyna Duda, Agata Wieczorkiewicz-Kabut, Anna Koclęga, Patrycja Zielińska, Krzysztof Woźniczka, Helena Krzemień, Anna Armatys, Grzegorz Helbig

**Affiliations:** Department of Hematology and Bone Marrow Transplantation, Faculty of Medicine in Katowice, Medical University of Silesia, Katowice, Poland

**Keywords:** Acute myeloid leukemia, Allogeneic stem cell transplantation, Complications, Elderly, Outcome

## Abstract

The incidence of AML increases with age. The implementation of reduced intensity conditioning and progress in supportive care enabled to perform allo-HSCT in elderly patients. The main objective of the study was to assess the safety and efficacy of allotransplantation in elderly AML.Forty nine patients (33 males) at median age of 68 years were identified. Data on patients’ and transplant’s related variables were retrieved from our local transplant registry. Most patients (65%) were transplanted from 10/10-HLA or 9/10-HLA matched unrelated donor, seven patients (14%) received stem cells from matched related donor and ten patients (20%) from haploidentical donor. All patients received reduced-intensity conditioning (RIC). Peripheral blood was a source of stem cells in all patients except one (98%). Acute GVHD developed in 22 patients (44%) with 5 individuals presenting grade III-IV. CMV reactivation was demonstrated in 19 patients (39%) till day + 100. In total, 22 patients (45%) have died. The main causes of death included infectious complications (*n* = 9), relapse with subsequent chemotherapy resistance (*n* = 7), steroid-resistant GvHD (*n* = 4) and other causes (*n* = 2). Twenty-seven patients (55%) were alive at the last contact, presented full donor chimerism and remained in the complete remission. The probability of OS and relapse-free survival (RFS) were 57% and 81% at 2 years, respectively. Older donor age showed negative impact on relapse. CMV reactivation, the severity of acute graft versus host disease and older donor age negatively influenced survival. Allo-HSCT remains a safe, feasible and effective procedure for elderly AML patients.

## Introduction

Acute myeloid leukemia (AML) is the most common type of acute leukemia among adults, mainly affecting older people with a median age at diagnosis of 68 years [1]. The prognosis of elderly patients with AML is dismal due to more frequent presence of poor predictive factors, both disease-related (such as adverse-risk cytogenetics, multidrug resistance and secondary leukemia) and patient-related (i.e. poor clinical condition, severe comorbidities, less tolerance to intensive therapy) [2, 3]. The standard induction treatment for fit elderly patients (> 60 years) consists of an anthracycline in combination with cytarabine usually at lower doses if compared with younger patients (< 60 years). For unfit elderly patients therapeutic options included less-intensive treatment—hypomethylating agents (azacitidine and decitabine) or low-dose cytarabine, however the responses were short-term. Nowadays, the development of novel agents and/or targeted therapy (e.g. venetoclax with azacitidine, ivosidenib plus azacitidine) improved response rate and prolonged survival [4].

Allogeneic hematopoietic stem cell transplantation (allo-HSCT) remains the only curative treatment for patients with AML, especially for those with intermediate- and high risk disease according to the European Leukemia Network (ELN) classification or favorable-risk patients with persistent measurable residual disease (MRD) [4, 5]. In the past, older patients were not considered as candidates for allo-HSCT due to high transplant related mortality. However, advances in supportive care, better donor selection and the development of reduced intensity conditioning (RIC) extended the indications for allo-HSCT to wider patient population including elderly one [6].

In this retrospective study, we present our institutional experience with allo-HSCT for acute myeloid leukemia in patients ≥ 65 years at transplant.

## Material and methods

The diagnosis of AML and response criteria were based on the European Leukemia Net recommendations [4, 7, 8]. European Group for Blood and Marrow Transplantation (EBMT) risk score was calculated as described by Gratwohl [9]. The hematopoietic cell transplantation-comorbidity index (HCT-CI) scores were assessed according to Sorror et al. [10]. Bone marrow evaluation was performed before conditioning, then on days + 30, + 60, + 100 after transplantation, then every 6 months. Donor chimerism was assessed from bone marrow and/or peripheral blood using short tandem repeat (STR) analysis. Acute and chronic graft versus host disease (GvHD) was assessed according to standardized terminology [11]. All patients provided an informed consent before the procedure.

## Statistics

To estimate the survival function from lifetime data, i.e. relapse and death after transplantation, the Kaplan–Meier’s (KM) estimator was used. A p-value < 0.05 was considered statistically significant and *p* < 0.1 as on the border of the statistical significance. The following variables were included in analysis: patient-related data (age, gender, ELN risk, blast proportion in blood and marrow, hemoglobin concentration, platelet count, neutrophil count, LDH activity), transplant-related data (disease status at transplant, MRD status, HCT-CI, EBMT score, donor’s age, donor and recipient gender, CMV status, ABO blood group, HLA compliance, the presence and grading of acute and chronic GVHD, the number of transplanted CD34 + and CD3 + cells). The cumulative incidence function (CIF) was used to analyze competing event data. In our analysis, to compare two groups with respect to failures for one event type of competing events, the Gray’s test was used [12]. The computation was performed in the R statistical platform [13].

## Results

### Patient characteristics

Forty-nine patients (33 males and 16 females) with AML at median age of 68 years at transplant underwent allo-HSCT between years 2012 and 2021. Thirty-three (67%) patients were diagnosed with de novo AML while 11 (22%) progressed from MDS, 2 patients (4%) progressed from chronic myelomonocytic leukemia and 3 (6%) had therapy-related AML (prior chemotherapy due to colon, lung and endometrial cancer). Cytogenetics on bone marrow cells were conclusive in 36 individuals: 18 patients demonstrated normal karyotype, 7—cytogenetic abnormalities not classified as favorable/adverse, 6 -complex karyotype, 3 -adverse cytogenetic [t(3;3), -7] and 2 -favorable cytogenetics [t(8;21, inv(16)]. Data on ELN risk stratification were available for 40 patients—6 (15%) of them showed favorable risk category, 20 (50%) intermediate and 14 (35%) high. Forty-seven patients received at least one anthracycline and cytarabine-based induction chemotherapy. A hypomethylating agent (azacitidine) was administered as the only therapy for two patients. 33 individuals (67%) achieved a complete remission (CR) after first induction therapy, eight (16%) after reinduction chemotherapy. Two patients achieved partial remission and six individuals had refractory/relapsed disease. Induction (anthracycline with cytarabine) and consolidation (cytarabine 2 cycles) were treated as first-line treatement (one line of therapy). Induction, re-induction with or without prior consolidation were treated as 2 lines of therapy. Induction, re-induction ± consolidation, and one or more salvage regimens were treated as ≥ 3 lines. No targeted therapies were used. Patient characteristics are summarized in Table 1.

**Table 1:** Patients’ characteristics

Variable	*n* = 49
Gender (female/male)	16/33
Age at diagnosis, years; median (range)	67 (63–73)
AML; n	
de novo	33
sAML	13
tAML	3
Bone marrow blasts at diagnosis, %; median (range)	55.2 (22–100)
LDH at diagnosis,	329
IU/L, median (range)	(146–1421)
Cytogenetics; n	
Favorable	2
Intermediate	25
Adverse	9
Missing	13
ELN risk stratification, n	
Favorable	6
Intermediate	20
Adverse	14
Missing	9
Treatment for AML; n	
HMAs	10
AML-like-induction	47
Number of lines of therapy before Tx; n	
1	26
2	19
≥ 3	4

AML = acute myeloid leukemia; sAML = secondary AML (after MDS/MPN), tAML = therapy-related AML; ELN = European LeukemiaNet; HMAs = The hypomethylating agents; Tx = transplantation

### Transplant data

#### Baseline characteristics of transplanted patients

Median recipient age was 68 years (range 65–73) whereas donors were younger—median age was 32 years (range 18–70). HCT-CI and EBMT score were calculated in all patients. The majority of patients had low (53%) or intermediate (24%) HCT-CI score, the similar results were noted for EBMT score: low 65% and intermediate 12%. Most patients (65%) were transplanted from 10/10-HLA or 9/10-HLA matched unrelated donor, seven patients (14%) received stem cells from matched related donor and ten patients (20%) from haploidentical donor. Peripheral blood was a source of stem cells in all patients except one (98%). All patients received reduced-intensity conditioning (RIC) based on treosulfan/fludarabine (*n* = 23, 47%), busulfan/fludarabine (*n* = 14, 29%) or other (*n* = 12, 24%). Anti-thymocyte globulin (thymoglobulin) at total dose of 6.0–7.5 mg was given for unrelated transplantations (*n* = 32) and for 3 patients who received graft from female matched related donor. For GVHD prophylaxis for all patients except those who received grafts from haploidentical donors cyclosporine with methotrexate (*n* = 23), cyclosporine with mycophenolate mofetil (*n* = 10) and tacrolimus with mycophenolate mofetil (*n* = 6) were given. Haploidentical transplants were performed with pos-transplantation cyclophosphamide, tacrolimus and mycophenolate mofetil (*n* = 10).

#### Outcome of transplanted patients

All patients except 2 who died early after allo-HSCT engrafted after median of 19 days (range 12–29). A platelet count greater than 20 × 10^9^/l was achieved after median of 14 days (range 9–25). Donor chimerism was achieved in 47 patients at discharge. Acute GVHD developed in 22 patients (44%) with 5 individuals presenting grade III-IV. Thirteen patients (27%) had chronic GvHD.

CMV reactivation was demonstrated in 19 patients (39%) till day + 100. CMV reactivation occurred more often in seropositive recipients (R +) receiving a graft from seronegative donors (D-) compared to R + D + [12/17 (71%) vs 7/31 (23%]. For those who reactivated, valganciclovir was implemented. There were two deaths until day + 30 after transplant – both due to pneumonia with septic shock. Two patients died of steroid-resistant acute GVHD between days + 30 and + 100 after transplantation. In total, 22 patients (45%) have died. The main causes of death included infectious complications – pneumonia, C. difficile infection, sepsis (Enterococcus spp., Escherichia coli, Klebsiella pneumoniae) (*n* = 9), relapse with subsequent chemotherapy resistance (*n* = 7), steroid-resistant GvHD (*n* = 4) and other—sudden cardiac arrest (*n* = 2). Twenty-seven patients (55%) were alive at the last contact, presented full donor chimerism and remained in the complete remission. The use of ATG was not associated with an increased rate of any infection when compared to patients who did not receive ATG [14/35 (40%) vs 10/14 (71%)]. Transplant data are summarized in Table 2.

**Table 2:** Transplant data

Variable	*n* = 49
Age of recipient, median; years (range)	68 (65–73)
Age of donor, median; years (range)	32 (18–70)
Disease status at transplant; n	
CR1	33
CR ≥ 2	8
Other (PR, refractory and relapsed)	8
EBMT score; n	
low	32
intermediate	6
high	11
HCT-CI score; n	
low	26
intermediate	12
high	11
Donor type; n	
10/10-HLA MRD	7
10/10-HLA URD	22
9/10-HLA URD	10
HID	10
Donor sex; n	
female	11
male	38
CMV status; n	
D + /R +	31
D-/R +	17
D + /R-	0
D-/R-	1
Graft source	
peripheral blood	48
bone marrow	1
Hemoglobin level (g/dL); median (range)	12.2 (7–15.5)
Neutrophil count (× 10^9^/L); median (range)	2.42 (0.21–9.16)
Platelet count (× 10^9^/L); median (range)	190 (10–611)
Blasts in bone marrow (%); median (range)	3.2 (0.5–17.5)
Conditioning regimen, n	
Treosulfan/Fludarabine	23
Busulfan/Fludarabine	14
Other	12
GVHD prophylaxis, n	
Cyclosporine with methotrexate	23
Cyclosporine with mycophenolate mofetil	10
Tacrolimus with mycophenolate mofetil	6
Tacrolimus, mycophenolate mofetil and post Cy	10
Number of transplanted CD34-positive cells (× 10^6^/kg); median (range)	5.76 (1.17–8.37)
ANC > 0.5 (× 10^9^/L); days, median (range)	19 (12–29)
PLT > 20 (× 10^9^/L); days, median (range)	14 (9–25)
Acute GvHD, n	
grade I-II	17
grade III-IV	5
Chronic GvHD, n	13

ANC = absolute neutrophil count; CMV = cytomegalovirus; CR = complete remission; Cy = cyclophosphamide; D = donor; R = recipient; EBMT = European Blood and Bone Marrow Transplantation; GvHD = graft versus host disease; HCT-CI = hematopoietic cell transplant comorbidity index; HID = haploidentical donor; MRD = matched related donor; PLT = platelet count; PR = partial remission; URD = unrelated donor

**Fig. 1:**
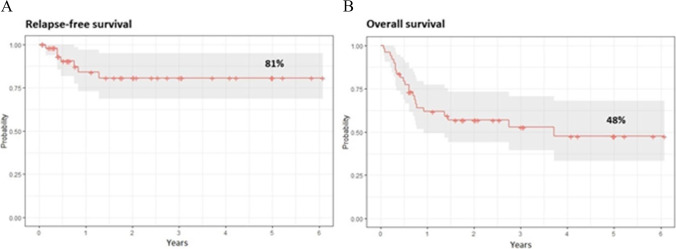
Kaplan–Meier’s survival curves showing relapse-free survival (**A**) and overall survival (**B**) after transplantation

**Fig. 2:**
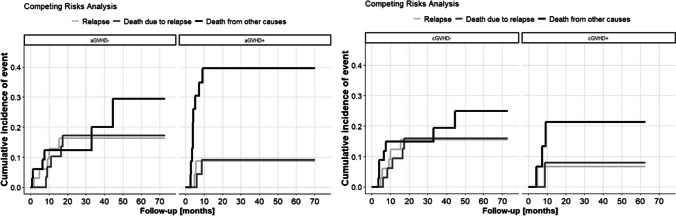
The competing risk analysis according to acute and chronic GVHD occurrence for relapse, death due to relapse, and death from other causes

For the analyzed clinical events (relapse & death), the survival curves are shown in Fig. 1 (A and B, respectively). The KM estimates of survivals in patients at 1, 2 and 5 years after transplantation are given in Table 3. The probability of OS and relapse-free survival (RFS) were 57% and 81% at 2 years, respectively. Based on the collected dataset, the statistically significant (*p* < 0.05) and those on the border of the statistical significance (*p* < 0.1) hazard ratios (HRs) with 95% confidence intervals (95% CI) and p-values were reported in Table 3. Relapse was nearly five times more common in patients who received stem cells from female donors, however, the estimate is on the border of the statistical significance (*p* < 0.1). The older the donor was, the greater was the risk of relapse (donor age was statistically significant – *p* < 0.05). Figure 2 presents the competing risk analysis according to aGVHD and cGVHD occurrence. There was no influence of aGVHD on relapse (HR = 0.57; 95% CI: 0.11—2.88; *p* = 0.50), death due to relapse (HR = 0.58; 95% CI: 0.11—2.96; *p* = 0.51), and death from other causes (HR = 2.39; 95% CI: 0.85—6.68; *p* = 0.10). Similarly, no impact of cGVHD on relapse (HR = 0.48; 95% CI: 0.06—4.17; *p* = 0.51), death due to relapse (HR = 0.48; 95% CI: 0.06—4.04; *p* = 0.50), and death from other causes (HR = 1.12; 95% CI: 0.30—4.15; *p* = 0.87) was demonstrated.

**Table 3:** Factors influencing post-transplant outcome

Outcome	Result (%/HR)	*p-*value
Relapse		
Donor sex (female vs. male)	4.60 (95% CI: 0.77–27.3)	0.0928
Donor age	1.05 (95% CI: 1.001–1.095)	0.0481
Death		
CMV reactivation	3.28 (95% CI: 1.05–10.2)	0.0402
aGVHD grade	2.08 (95% CI: 1.34–3.24)	0.0048
Donor sex (female vs. male)	3.07 (95% CI: 0.89–10.6)	0.0753
Donor age	1.04 (95% CI: 1.01–1.07)	0.0048

**Fig. 3:**
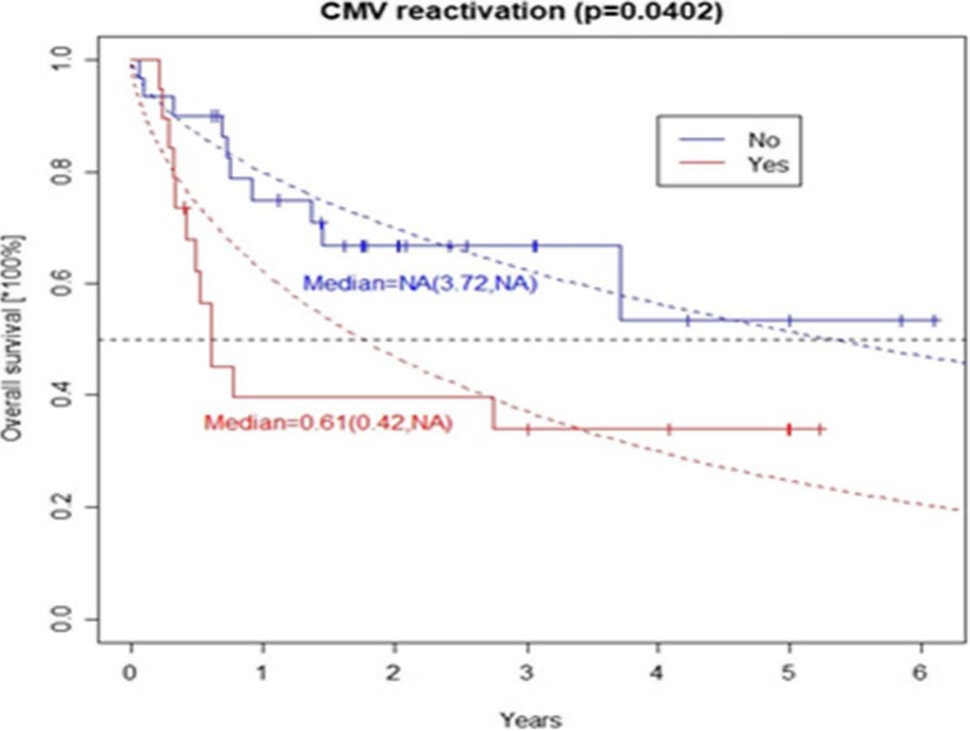
Overall survival in patients with or without CMV reactivation after transplantation

**Fig. 4:**
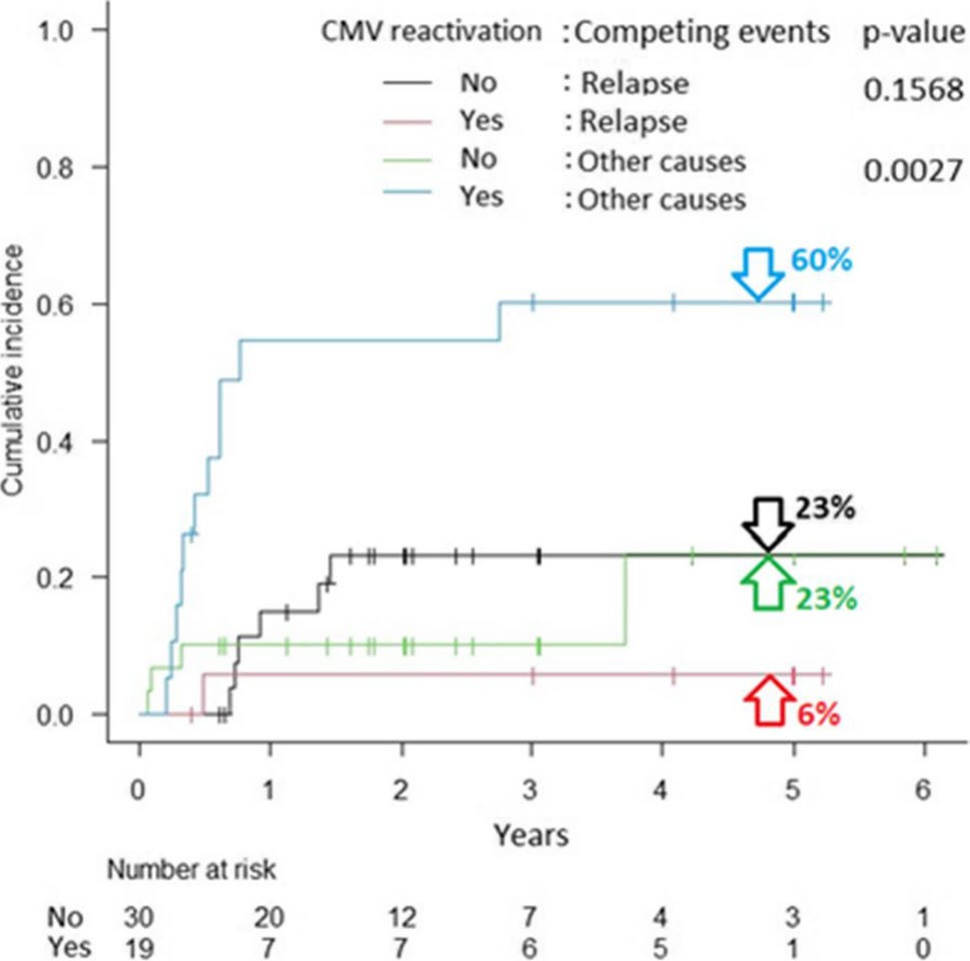
Cumulative incidence functions for death due to relapse or other causes in patients without or with CMV reactivation (together with Gray’s test p-values)

**Fig. 5:**
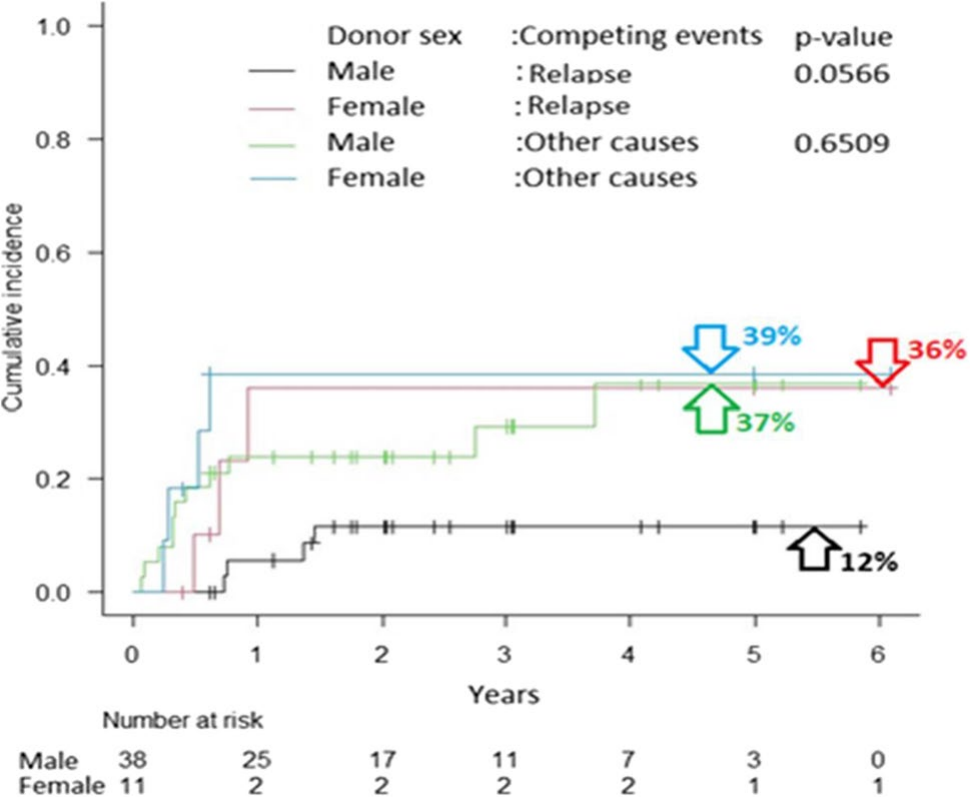
Cumulative incidence functions for death due to relapse or other causes by donor sex (together with Gray’s test p-values)

CMV reactivation after transplantation induced over three-fold increase in the risk of patient death. The grade of acute graft versus host disease (aGVHD), female gender (however, with a p-value < 0.1) and age (at *p* < 0.05) of the donor influenced survival (Table 3, for details). The descending curves (with median survivals) following the KM estimator for CMV reactivation variable were plotted in Fig. 3. The estimated CIFs for death due to relapse or other causes together with Gray’s tests between the patients without or with CMV reactivation, and donor gender effect are displayed graphically in Figs. 4 and 5. Disease status at transplant (hematologic and MRD), EBMT score as well as HCT-CI did not have an impact on post-transplant outcomes. Moreover, donor age and gender as well as gender combination between donor and recipient did not influence the outcome.

## Discussion

The prognosis of elderly AML patients is dismal and allo-HSCT remains the only chance to improve their outcome. In our study, the estimated 2-year OS was 57% and our results were better to those reported by others [6, 14–19]. In a large American study (reported by Center for International Blood and Marrow Transplant Research – CIBMTR) the 2-year OS of 1106 patients aged 70 years and older mostly with AML (54%) transplanted from 2000 to 2013 was merely 36%. Overall survival improved significantly over time (from 26% in 2000–2007 to 39% in 2008–2013) [6]. In other large study (713 patients age ≥ 70 with AML reported by European Society for Blood and Marrow Transplantation—EBMT) survival at 2 years was 38% [18]. The 2-year OS in another American analysis was 30.7%, with median age of included patients of 69 years [19] and the results were in line with those reported by Brunner et al.: OS at 2 years was 39% [16]. Better results were presented by other study groups with 2-year OS of 46% [13]. In other study patients receiving allo-HSCT in CR1 were compared with those treated with chemotherapy only – a significant improvement in OS was demonstrated for the former group: 77.5% vs 27.3 at 3 years, however only 17 of 121 patients in CR1 were proceeded to HSCT [20]. Unsuccessful results were presented in a population-based study from the Netherlands (1989–2012) – a 5-year relative survival rates were 14% and 2% among AML patients 61–70 and > 70 years, respectively. No patient over the age of 70 years was allografted [21]. Current data strongly suggest that elderly patients can benefit from allo-HSCT when compared with those treated with chemotherapy only.

We analyzed impact of pretransplant and transplant-related factors affecting patients’ survival. In our study older donor age affected survival; the impact was evident both on relapse and death. Similar results were presented elsewhere—an allograft from a donor over 50 years old was associated with significantly lower outcome [22, 23]. By contrast, it was demonstrated that grafts from donors ≥ 60 years old do not adversely affect outcomes compared with grafts from younger donors [24]. Our results showed that female donors had a negative impact on relapse and OS. By contrast, Kim et al. found that female donors for male recipients were associated with decreased incidence of relapse [25]. Other large registry also reported that transplantation from a female donor for male recipient was associated with a lower relapse rate [26].

In our study we also demonstrated that CMV reactivation had a negative impact on OS. These findings are in agreement with some previous studies [27, 28]. Despite higher NRM in patients with CMV reactivation, the incidence of relapse was lower and similar results were reported by other authors in single-center studies [29, 30]. Some mechanisms of the anti-leukemic effect of cytomegalovirus were postulated—direct effect of virus on AML cells, immune response inducing the proliferation of NK and T cells (NK cell–mediated “graft-versus-leukemia” effect, T cell-mediated cytotoxicity) [29, 30]. By contrast, in large registry study (9469 patients from the CIBMTR database) CMV reactivation was not associated with reduced relapse rate [31].

Another factor that influenced OS was the occurrence of grade III-IV acute GVHD and this finding is in line with data presented by other authors [6, 18]. Infections remained the most frequent cause of death in our patients (41%), 18% died from steroid-resistant-GVHD and 32% due to leukemia relapse. Of note is that disease relapse/progression was the commonest cause of death in reports presented by other groups [6, 18, 19].

Despite the relatively small number of patients, our analysis showed that allo-HSCT may be an effective and safe treatment option for older patients with AML. Our study has also some limitations. This report was a retrospective analysis and included data from a single institution registry, patients' population was heterogenous and not all data were available. Moreover some results were insignificant due to the small study group.

The number of allogeneic transplants in elderly has increased over the last years, however not all patients are good candidates for this procedure. It is predicted that the proportion of elderly AML patients who may proceed to allo-HSCT will increase in the next years. Of note is that accepting such patients for transplantation requires careful evaluation focusing on performance status, every-day function with the assessment of cognitive impairment. A multidisciplinary team should also be involved if needed. The achievement of remission at transplant seems to be one of the most important factor determining the success of transplantation, however it was not a case for our analysis. However, one should bear in mind, that majority of our patients were transplanted in complete remission (41/49) and only 8 patients remained in partial response or active disease. Despite lack of severe co-morbidities, none of the patients received myeloablative regimen. On the other hand, one should keep in mind that median age at transplant was 68 years. The optimal reduced-intensity conditioning for elderly with AML is a still matter of debate and preparative regimen should be individually tailored. Despite an advanced age, most transplanted patients had low HCT-CI and EBMT scores and procedure was found to be relatively safe. Of note is that none of the patients received post-transplantation maintenance which remained a current standard especially for those with MRD positivity or high-risk pretransplant features. Nevertheless, the long-term outcome of the patients is encouraging.

## Conclusions

Allogeneic hematopoietic stem cell transplantation is a feasible procedure for elderly AML patients with promising results, but careful individualized evaluation of risk–benefit ratio is required.
